# Effects of Ferumoxides – Protamine Sulfate Labeling on Immunomodulatory Characteristics of Macrophage-like THP-1 Cells

**DOI:** 10.1371/journal.pone.0002499

**Published:** 2008-06-25

**Authors:** Branislava Janic, A. S. M. Iskander, Ali M. Rad, Hamid Soltanian-Zadeh, Ali S. Arbab

**Affiliations:** Cellular and Molecular Imaging Laboratory, Department of Radiology, Henry Ford Hospital, Detroit, Michigan, United States of America; Albert Einstein College of Medicine, United States of America

## Abstract

Superparamagnetic Iron Oxide (SPIO) complexed with cationic transfection agent is used to label various mammalian cells. Labeled cells can then be utilized as an *in vivo* magnetic resonance imaging (MRI) probes. However, certain number of *in vivo* administered labeled cells may be cleared from tissues by the host's macrophages. For successful translation to routine clinical application of SPIO labeling method it is important that this mode of *in vivo* clearance of iron does not elicit any diverse immunological effects. The purpose of this study was to demonstrate that SPIO agent ferumoxides-protamine sulfate (FePro) incorporation into macrophages does not alter immunological properties of these cells with regard to differentiation, chemotaxis, and ability to respond to the activation stimuli and to modulate T cell response. We used THP-1 cell line as a model for studying macrophage cell type. THP-1 cells were magnetically labeled with FePro, differentiated with 100 nM of phorbol ester, 12-Myristate-13-acetate (TPA) and stimulated with 100 ng/ml of LPS. The results showed 1) FePro labeling had no effect on the changes in morphology and expression of cell surface proteins associated with TPA induced differentiation; 2) FePro labeled cells responded to LPS with slightly higher levels of NFκB pathway activation, as shown by immunobloting; TNF-α secretion and cell surface expression levels of CD54 and CD83 activation markers, under these conditions, were still comparable to the levels observed in non-labeled cells; 3) FePro labeling exhibited differential, chemokine dependent, effect on THP-1 chemotaxis with a decrease in cell directional migration to MCP-1; 4) FePro labeling did not affect the ability of THP-1 cells to down-regulate T cell expression of CD4 and CD8 and to induce T cell proliferation. Our study demonstrated that intracellular incorporation of FePro complexes does not alter overall immunological properties of THP-1 cells. The described experiments provide the model for studying the effects of *in vivo* clearance of iron particles *via* incorporation into the host's macrophages that may follow after *in vivo* application of any type of magnetically labeled mammalian cells. To better mimic the complex *in vivo* scenario, this model may be further exploited by introducing additional cellular and biological, immunologically relevant, components.

## Introduction

Over the recent years imaging techniques that enable efficient and non-invasive *in vivo* monitoring and trafficking of transplanted cells have become central to the successful development of cell transplantation based diagnostic and therapeutic approaches. Currently, a number of imaging modalities, such as positron emission tomography (PET), single photon emission-computed tomography (SPECT) and magnetic resonance imaging (MRI) are being perfected for the purpose of *in vivo* cell tracking. Nevertheless, translation to routine clinical application of such methods strongly depends on the availability of efficient cell labeling reagent that does not exhibit toxic effect in labeled cell, enables successful detection by chosen imaging technique and most importantly, does not elicit side effects in human recipients.

One of the contrast agents used with MRI imaging technique is superparamagnetic iron oxide (SPIO). SPIO has been in use as an intravenous MRI contrast agent for analyzing the liver pathology [1], but also as an *ex vivo* cell labeling agent for various types of mammalian cells that when labeled can be utilized as an *in vivo* MRI probes. For example, SPIO has been successfully used for the labeling of cancer cells [2], T cells [3], dendritic cells [4] and stem cells [5], [6]. As reported before, SPIO labeling does not exhibit adverse effects on cell physiology [7] and when combined with cationic transfection reagents, is relatively stably incorporated within the endosomal cellular compartment [8]. Regardless of the mode of *in vivo* SPIO administration, intravenously or within the labeled cells, its application is coupled with time dependent change in the MRI signal intensity due to the *in vivo* dilution of administered iron that is attributed to the clearance of iron by host immune cells. When administered intravenously as free circulating SPIO nanoparticles, most of the nanoparticles are cleared by the resident phagocytic cells in liver (Kupffer cells) and spleen (splenic macrophages) [1], [9]. When administered as intracellularly incorporated iron particles, the most probable mode of SPIO release into the extracellular space is exocytosis that may be coupled with cell division [10]. The released SPIO particles may ultimately be cleared from tissues by resident/tissue macrophages. In addition, certain number of *in vivo* administered labeled cells undergoes apoptosis and these dead cells may also be cleared from tissues by host macrophages. The effect of this iron load on the functional properties of host macrophage system has not been studied before. For successful clinical application of SPIO labeling method it is important that this mode of *in vivo* clearance of iron or FePro labeled cells does not elicit any diverse immunological effects.

Previously, we have reported a novel method for generating magnetically labeled cells that is based on combining Ferumoxides (Fe) and Protamine Sulfate (Pro) into Superparamagnetic Iron Oxide (SPIO)-transfection agent complex (**FePro**) [6], [11]. Ferumoxide is a suspension of dextran coated SPIO particles that has been approved by US Food and Drug Administration (FDA) as an *in vivo* MRI contrast reagent for the use in humans. Protamine Sulfate is a polycationic agent (also approved by FDA for clinical use) that when combined with Fe enables efficient and effective intracellular incorporation of SPIO particles. In this study we utilized THP-1 cell line to demonstrate that FePro incorporation into macrophages does not alter immunological properties of these cells. THP-1 cells, a human monocytic cell line, were derived from the patient with acute monocytic leukemia. THP-1 cells were described to have characteristics of monocyte cell type and upon stimulation with reagents such as phorbol esters or 1,25 dihydroxy vitamin D_3_, can be differentiated into immunologically active cells, i.e. macrophages [12]. THP-1 cell line has been extensively characterized [13], [14], [15] and widely used as a model for studying macrophage cell type [16], [17], [18]. In addition, due to the good labeling efficiency and reproducibility, THP-1 cells have very often been a cell type of choice for characterizing the SPIO labeling agents [19], [20]. We hypothesize that FePro labeling will not affect the physiological ability of THP-1 cells to maintain non-differentiated state nor it will affect their ability to respond to the activation stimuli and to modulate T cells response.

## Materials and Methods

### Cell culture

THP-1 cells (ATCC, Rockville, MD) were maintained in RPMI 1640 (Sigma, St. Louis, MO) supplemented with 10% heat inactivated Fetal Calf Serum (Hyclone) and 0.05 mM 2-mercaptoethanol (Chemicon), as recommended by ATCC. For each experiment cells were labeled with Fe-Pro (described in detail in the section bellow), incubated for 24 h in the presence of 100 nM of phorbol ester, 12-Myristate-13-acetate (TPA) (Cell Signaling Tecnology) to induce differentiation. Following the differentiation, cells were stimulated for 30 min or 4 h with 100 ng/ml of *Escherichia coli* lipopolysaccharide (LPS).

### Cell labeling

THP-1 cells were labeled with ferumoxides-protamine sulfate complex (Fe-Pro) according to our previously reported method [6], [11]. In brief, FePro was generated by mixing the commercially available ferumoxides (Feridex IV; Berlex Laboratories, Wayne, NJ, USA) and protamine sulfate (American Pharmaceuticals Partners, Shaumburg, IL, USA). Protamine sulfate supplied as 10 mg/ml of stock solution was freshly diluted to a concentration of 1 mg/ml in distilled water at the time of use. Ferumoxides was suspended in RPMI 1640 medium (Mediatech, Herndon, VA, USA) supplemented with MEM nonessential amino acids and sodium pyruvate, at the concentration of 100 µg/ml. Protamine sulfate was then added to the ferumoxide suspension to the final concentration of 4.5 µg/ml. Ferumoxides and protamine sulfate mixture was intermittently mixed for about a minute to generate Fe-Pro complex. THP-1 cells were washed once and suspended at the concentration of 4×10^6^ cell/ml in RPMI containing the Fe-Pro complex, and incubated for 2 hours within the 6-well culture plates at 37°C, in 5%CO_2_ humidified atmosphere. After 2 hours of incubation complete THP-1 media was added to adjust the concentration of cells and Fe-Pro to the following: 2×10^6^ cells/ml for THP-1 cells, 50 µg/ml for ferumoxides/mL and 2.25 µg/ml for protamine sulfate. After overnight incubation at 37°C in 5%CO_2_ humidified atmosphere, cells were washed three times with 1×PBS to eliminate extracellular Fe-Pro complexes and used for the experiments.

### Staining for intracellular iron and determining the labeling efficiency

Aliquots of labeled and non-labeled control THP-1 cells were transferred to glass slides and fixed with 4% paraformaldehyde. Prussian blue cellular staining was performed by incubating fixed cells in a mixture of 4% potassium ferrocyanide and 3.7% hydrochloric acid (Perl reagent for Prussian blue staining) for 30 minutes. Slides were then washed and cells counterstained with nuclear fast red. The cells were evaluated by light microscopy to asses the labeling efficiency. Cells exhibiting blue intracellular particles were considered Prussian-blue positive. The percentage of labeled cells was determined from the average of the labeled cells to the total number of cells from four randomly selected fields of view.

### Intracellular Iron Quantification

Quantification of intracellular iron in THP-1 labeled cells was performed as previously described [11], [21]. Briefly, after labeling with FePro, the cells were washed three times with 1×PBS to dispose of extracellular FePro. Triplicates of labeled and non-labeled THP-1 cells (2×10^5^ cells per tube) were centrifuged at 3000 rpm for 5 minutes. After discarding the supernatants, cell pellets were incubated at 110°C overnight (no cap on tubes). The next day, iron was dissolved by adding 1 mL of 5 M hydrochloric acid to each tube and samples were further incubated at 60°C for 4 h. During this incubation step, tubes were capped to prevent acid evaporation. Then, 0.5 ml of acid solution from each tube was transferred to a separate 1.5 ml cuvette and absorbance was read at 351 nm. The average absorbance value for each sample (3 tubes for each sample) was divided by the number of cells to determine the average iron concentration per cell. Iron concentration was determined using the standard curve obtained by plotting the known iron concentration of several dilutions of iron vs. absorbance.

### TNF-α protein secretion – ELISA

After labeling with Fe-Pro complex, THP-1 cells were suspended in complete THP-1 cell media at the concentration of 1×10^6^ cells/ml and differentiated for 24 h in the presence of 100 nM of TPA (at 37°C in 5% CO_2_). After cell differentiation, culture media was replaced and cells were incubated for additional 4 hours in the presence of 100 ng/ml of LPS. After LPS stimulation, THP-1 cell supernatants were collected and analyzed for the levels of secreted TNF-α protein by ELISA MAX™ Set *Deluxe* kit (BioLegend) according to the manufacturer's instructions. Reference curve for each experiment was obtained by plotting the TNF-α concentration of several dilutions of standard protein vs. absorbance.

### Nuclear and cytoplasmic protein extraction

Nuclear and cytoplasmic protein extracts were prepared using the Nuclear Extraction Kit (Active Motif, Carlsbad, CA) according to the manufacturer's instructions. In brief, cell pellets were re-suspended in 1× Hypotonic Buffer (Active Motif), and incubated on ice for 15 minutes. Detergent was then added and the suspension was centrifuged for 30 seconds at 14,000×*g* in a microcentrifuge pre-cooled to 4°C. Supernatants containing the cytoplasmic protein fraction were collected, while the nuclear pellets were re-suspended in Complete Lysis Buffer (Active Motif), vortexed for 10 seconds and incubated 30 minutes on ice on a rocking platform at 150 rpm; vortexed for 30 seconds at the highest setting and centrifuged for 10 minutes at 14,000×*g* in a microcentrifuge pre-cooled to 4°C. The nuclear fractions were collected into a pre-chilled microcentrifuge tubes. Protein concentration was determined with Bio-Rad Protein Assay (Bio-Rad Laboratories, Hercules, CA), using Bovine Serum Albumin (BSA) as a standard.

### Western blot - NFκB and IκB expression analysis

Five µg of nuclear protein and 10 µg of cytoplasmic protein were resolved on 10% SDS polyacrylamide gels, transferred to nitrocellulose membrane (BioRad) and probed overnight at 4°C with specific primary antibodies: mouse monoclonal IgG_3_ for p65 subunit of NF-κB (MAB3026) (Chemicon International) at the concentration of 5 µg/ml and 1∶500 dilution of rabbit polyclonal IgG for IκBα (624901) (Biolegend). Primary antibody binding was detected by incubating the membrane in the presence of horseradish peroxidase (HRP) conjugated secondary polyclonal donkey antibody with specificity for rabbit IgG or polyclonal sheep antibody with specificity for mouse IgG (both from GE Healthcare Lifesciences) for 1 h at room temperature. The signal was detected by enhanced chemiluminescence using the ECL Detection Kit (GE Healthcare Lifesciences).

### THP-1 cell migration assay

Rantes and Monocyte Chemotactic Protein-1(MCP-1) induced THP-1 cell directional migration was analyzed using QCM™ Chemotaxis 5 µm 96-well Cell Migration Assay kit (Chemicon) according to the supplier's experimental protocol. In brief, THP-1 cells were labeled with Fe-Pro and differentiated for 24 h with TPA, as described. After differentiation, cells were resuspended in serum free RPMI at the concentration of 2×10^6^ cell/ml and 100 µl of cell suspension was placed into migration chamber of QCM™ Chemotaxis Plate Assembly (Chemicon). Recombinant human Rantes and MCP-1 (BioLegend, San Diego, CA) were diluted in serum free RPMI to final concentrations of 50 and 300 ng/ml, respectively. Total of 150 µl of this solution was placed into the wells of the bottom, i.e. feeder tray. As suggested by manufacturer and as described before [22], in parallel experiment 150 µl of RPMI containing 10% FBS was loaded into the wells of the feeder tray, as a positive control. Chemotaxis plate was then re-assembled and incubated at 37°C in 5% CO_2_ for 4 hours to allow for THP-1 cells to migrate from the top to the bottom chambers through 5 µm size pores. After incubation, 75 µl of suspension containing cells that migrated was transferred from migration feeder tray to the well of a new 96-well plate; while the migrated cells that were still attached to the bottom side of the migration chamber filter were dislodged by incubating in Cell Detachment Solution (Chemicon) for 30 minutes (37°C in 5% CO_2_). Seventy five µl of dislodged cells were mixed with the cells that were previously collected from the migration feeder tray and incubated for 15 minutes with 50 µl of Lysis Buffer/Dye Solution (Chemicon) after which fluorescence was read using 480/520 filter set.

### THP-1 cell co-culture with T cells - CD4 and CD8 expression and THP-1 induced allogeneic T cell proliferation

To determine the effect of Fe-Pro labeling on THP-1 cells' ability to modulate T cells with regard to CD4 and CD8 expression and proliferation, co-culture was set up with the THP-1 cells/T cell ratio of 1∶10. T-cells were isolated the previous day from human cord blood, with an IRB approved protocol, by Ficoll gradient centrifugation followed by immunomagnetic CD3^+^ selection using MACS® system (Miltenyi Biotec Inc, Auburn, CA) according to the protocol supplied by the manufacturer. Isolated CD3^+^ cells were cultured in the presence of IL-2 cytokine (10 ng/ml) for 24 h, after which cells were analyzed by flow cytometry for the expression of CD3 and used in co-culture experiments. For the analysis of CD4 and CD8 expression on T cells, FePro labeled THP-1 cells were differentiated in the presence of 100 nM of TPA for 24 h in 24-well culture plates (5×10^5^ cells per well). After differentiation, THP-1 cells were stimulated with 100 ng/ml of LPS for 4 hours, after which media was replaced with the fresh media containing CD3^+^ T cells. THP-1 cells were co-cultured with CD3^+^ T-cells for 24 h at 37°C in 5% CO_2_. After 24 h of co-culture, cells were analyzed by flow cytometry for the expression of cell surface CD4, CD8 and CD3 markers. For the analysis cells were gated based on forward angle light scatter (FSC) and side angle light scatter (SSC) characteristics (R1) and the expression of CD3 marker (R2). To analyze the effect of FePro labeled THP-1 cells on T cell proliferative activity, TPA differentiated and γ-irradiated (20 Gy) THP-1 cells were co-cultured with T cells (1∶10 ratio). After 3 and 7 days of co-culture T cell proliferation was analyzed by performing MTT (3-[4,5-dimethylthiazol-2-yl] -2,5-diphenyl tetrazolium bromide) assay (Roche Molecular Biochemicals) according to the protocol supplied by the manufacturer. The absorbance of the formazan product was measured at a wavelength of 570 nm with 750 nm (subtracted) as reference.

### Flow cytometry

Fe-Pro labeled THP-1 cells were differentiated for 24 h in the presence of 100 nM of TPA and stimulated for 4 h with 100 ng/ml of *E.coli* LPS. Cells were washed in ice cold 1× PBS and incubated for 30′ on ice, in dark, with the respective fluorescently labeled antibodies. Fluorescence activated flow cytometry was performed with LSR II (Becton Dickinson) flow cytometer and a minimum of 10,000 events were analyzed for each sample. Live cells used for the analysis, were gated based on forward angle light scatter (FSC) and side angle light scatter (SSC) and further analyzed using the Cell Quest Pro software (Becton Dickinson).

Specific antibodies that were used in flow cytometric experiments to analyze the expression of cell surface markers were: FITC conjugated mouse anti-human CD3 IgG2b (Biolegend), PE conjugated mouse anti-human CD8 IgG1 (Biolegend), PE/Cy5 conjugated mouse anti-human CD4 IgG1 (Biolegend), PE conjugated mouse anti-human CD117 IgG1 (Biolegend), PE conjugated mouse anti-human CD54 IgG2a (Biolegend), FITC conjugated mouse anti-human CD11b IgG1 (Biolegend), PE/Cy5 conjugated mouse anti-human CD86 IgG2b (Biolegend), PE conjugated mouse anti-human CD83 IgG1 (BD Biscience), PE/Cy5 conjugated mouse anti-human HLA-DR IgG2a (Biolegend) and FITC conjugated mouse anti-human CD195 IgG2a (Biolegend). All the antibodies were used in the concentrations suggested by the suppliers.

### Statistical analysis

Each experiment was performed at least two times and each sample was tested in triplicate. Data are expressed as mean±SD. Statistically significant difference was determined by performing one way ANOVA analysis followed by Fisher's PLSD post-hoc test, when the there were more than two groups. For analysis between two groups student-t test was used. A value of p<0.05 was considered significant.

## Results

### Effect of Fe-Pro labeling on THP-1 cells ability to differentiate in response to TPA

**Figure 1:**
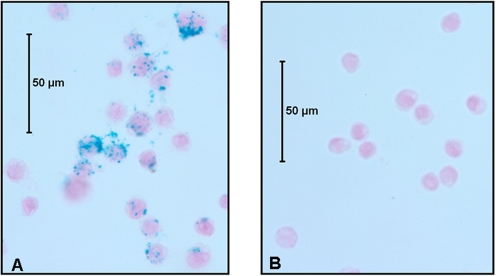
Prussian blue staining of FePro labeled THP-1 cells. THP-1 cells were labeled overnight with ferumoxide-protamine sulfate complex and stained using Prussian blue method to visualize the intracellular iron incorporation (A). Non-stained THP-1 cells were used as a negative control (B).

Labeling procedure and cellular incorporation of FePro complexes did not affect the ability of THP-1 cells to differentiate towards macrophage cell type. To confirm the FePro cellular uptake, THP-1 cells were stained with Prussian blue. Representative bright light photomicrographs of FePro labeled THP-1 cells are shown in Figure 1. Labeled THP-1 cells exhibited abundant heterogeneous uptake of FePro complexes that typically appears as blue granules inside the cytoplasm. Labeling efficiency analysis revealed that more than 95% of the cells were positive for Prussian blue. Quantitative analysis of intracellular iron concentration confirmed the microscopically observed THP-1 cell iron internalization. Iron labeled THP-1 cells contained an average of 8.2+0.30 pg iron/cell, versus 0.36+0.01 pg iron/cell in non-labeled THP-1 cells.

**Figure 2:**
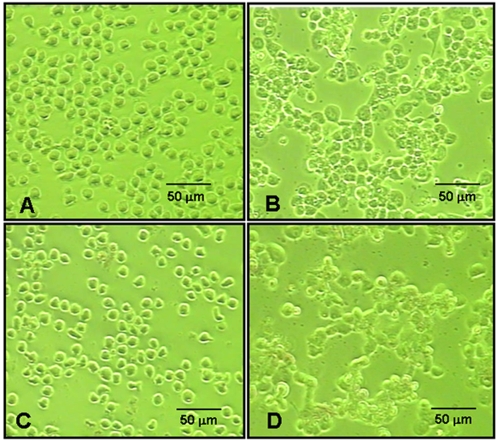
Effect of FePro labeling on THP-1 cell differentiation. THP-1 cells were labeled with FePro complexes and then differentiated in the presence of 100 nM of TPA for 24 h. Phase contrast photomicrograhs of non-labeled THP-1 cells before (A) and 24 h after incubation with 100 nM of TPA (B). FePro labeled THP-1 cell before (C) and 24 h after incubation with 100 nM of TPA (D).

After stimulation with 100 nM of TPA for 24 h, THP-1 cells underwent morphological changes that indicated differentiation towards macrophage cell type. As confirmed by phase contrast microscopy, through the process of differentiation monocyte like THP-1 cells changed from non-adherent, suspension cells to the macrophage like cells that exhibited cell adhesion, spreading, increased granularity and attachment to the bottom of the culture plates. There was no difference in the morphological appearance between FePro labeled and non-labeled THP-1 cells before or after TPA induced differentiation (Figure 2).

**Figure 3:**
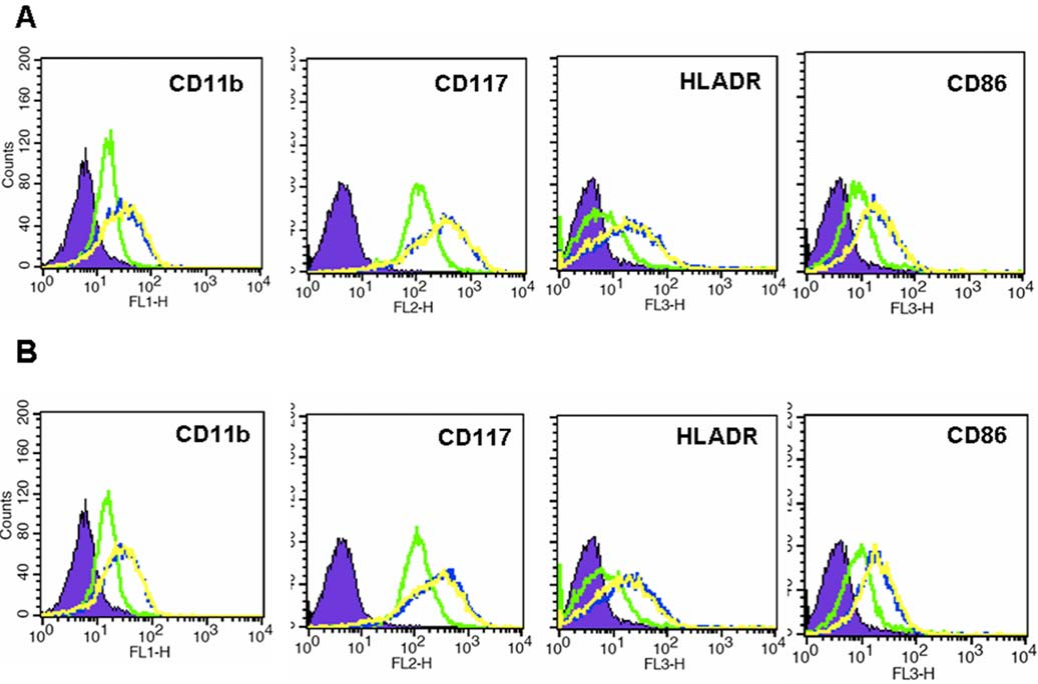
Effect of FePro labeling on the THP-1 cells expression of differentiation induced cell surface markers. The data depicts the levels of protein expression in non-labeled (A) and FePro labeled THP-1 cells (B) after 24 h incubation with 100 nM of TPA and following 4 h incubation with 100 ng/ml of LPS. After incubation, cells were stained with FITC conjugated anti-CD11b, PE conjugated anti-CD117 and PE-Cy5 conjugated anti-HLA DR and anti- CD86. Flow cytometric histograms of the non treated (control) cells (solid green lines), TPA treated (solid blue lines) and TPA and LPS treated (solid yellow lines) from one representative experiment are shown (n = 3). At least 10,000 live-gated cells were analyzed for FITC, PE or PE-Cy5 expression. Isotype control shown as solid blue histograms.

In addition to the morphological changes, differentiation process is associated with phenotypical changes that include changes in expression of specific cell surface proteins that play important roles in regulating immunological competence of macrophage type cells. To determine whether intracellular FePro incorporation affected the ability of THP-1 cells to upregulate cell surface proteins in response to TPA stimulation, we analyzed the levels of CD11b, CD117, HLA-DR and CD86 cell surface differentiation markers in FePro labeled and non-labeled THP-1 cells. No significant differences in the levels of CD11b, CD117, HLA-DR and CD86 expression between FePro labeled and non-labeled THP-1 cells were observed, as determined by flow cytometric analysis at 24 h of incubation in the presence of 100 nM of TPA (Figure 3).

### Effect of Fe-Pro labeling on THP-1 cells ability to respond to LPS stimulation

One of the best described macrophage activators commonly released by Gram-negative bacteria during the course of infection is LPS. To assess the ability of FePro labeled THP-1 cells to respond to LPS stimulation we have analyzed the effect of LPS on the expression levels of cell surface CD54 and CD83 activation markers, TNF-α secretion and activation of intracellular NFκB signaling pathway.

**Figure 4:**
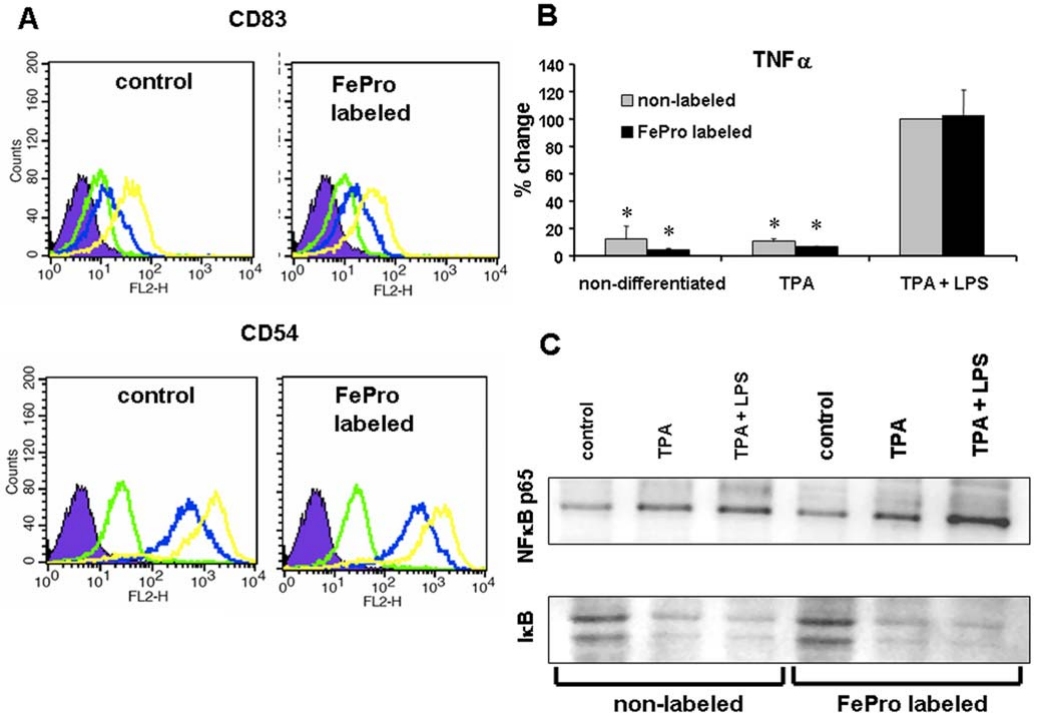
Effect of FePro labeling on THP-1 cell ability to respond to LPS stimulation. Effect of LPS on the levels of CD83 and CD54 cell surface expression (A), TNF-α production (B) and activation of NFκB signaling pathway (C) in non-labeled and Fe-labeled THP-1 cells. (A) After incubation with LPS, cells were stained with PE conjugated anti-CD54 and anti-CD83 antibodies. Flow cytometric histograms of the non treated (control) cells (solid green lines), TPA treated (solid blue lines) and TPA and LPS treated (solid yellow lines) from one representative experiment are shown (n = 3). At least 10,000 live-gated cells were analyzed for PE expression. Isotype control shown as solid blue histograms. (B) TNF-α protein levels in THP-1 cells supernatants after 4 h of stimulation with 100 ng/ml of LPS, determined by ELISA. Data expressed as means±SD. * p<0.05 compared to non-labeled, TPA differentiated and LPS stimulated control cells whose maximum response was set to be 100%. (C) Images of Western blots of nuclear protein fraction probed with anti- p65NFκB antibody (top panel) and cytoplasmic protein fraction probed with anti-IκBα antibody (lower panel). The data depicts the levels of protein in non-labeled and FePro labeled THP-1 cells after 24 h incubation with 100 nM of TPA and following 30 minutes with 100 ng/ml of LPS. Single representative experiment is shown (n = 3). The same patterns of relative NFκB and IκBα expression were observed in all three experiments.

Flow cytometric analysis of FePro labeled and non-labeled, TPA differentiated THP-1 cells showed that LPS stimulation increased the level of CD54 and CD83 expression in THP-1 cells. The levels of upregulated protein expression were comparable in FePro labeled and non-labeled cells (Figure 4A). In addition to the LPS induced changes at the THP-1 cell surface, we have also analyzed the effect of LPS on TNF-α cytokine expression. As determined by ELISA, after 24 h of TPA (100 nM) induced differentiation and 4 h of stimulation with 100 ng/ ml of LPS, no difference was observed (p>0.05) between non-labeled and FePro-labeled THP-1 cells in the levels of secreted TNF-α protein (Figure 4B).

Most of the biological effects of LPS on macrophage type cells have been extensively characterized and are achieved through the activation of NFκB cell signaling pathway [23]. Stimulation of this pathway results in the phosphorylation of cytoplasmic IκBα which then dissociates from NFκB enabling it to translocate to the nucleus and facilitate gene transcription. Dissociated IκBα is subsequently degraded and that results in a decrease of cytoplasmic IκBα levels. It has been shown that TNF-α as well as CD54 expression is regulated by NFκB [24]. To determine the effect of FePro labeling on NFκB signaling pathway we have performed Western blot analysis of cytoplasmic and nuclear protein fractions extracted from TPA differentiated THP-1 cells that were stimulated with 100 ng/ ml of LPS for 30 minutes. Immunobloting of the nuclear protein extract showed that FePro labeled as well as non-labeled cells up-regulated the levels of nuclear p65 NFκB protein in response to TPA. These levels were further increased upon the stimulation with LPS. It appeared that FePro labeled calls responded with slightly higher levels of nuclear p65 NFκB, as compared to the same in non-labeled cells (Figure 4C, top panel) and the same pattern of relative p65 NFκB expression were observed in repeated experiments (n = 3). However, this difference did not translate into discrepancies at the protein (TNF-α and CD54) levels between FePro labeled and non-labeled cells. As anticipated, parallel changes were observed in cytoplasm with regard to the levels of IkBα protein, i.e. TPA induced a decrease that was further potentiated with LPS stimulation. The observed decrease in IkBα expression levels was comparable in FePro labeled and non-labeled THP-1 cells and the same patterns of IkBα relative expression were seen in three separate experiments (Figure 4C, bottom panel).

### Effect of FePro labeling on THP-1 cells chemotaxis

**Figure 5:**
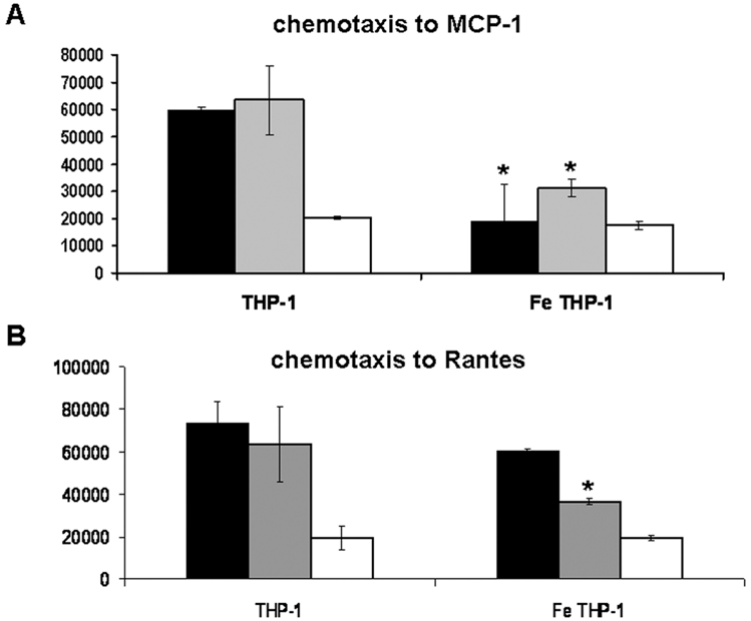
Effect of FePro labeling on THP-1 cell chemotaxis in response to MCP-1 and Rantes. THP-1 cells incubated in the presence of MCP-1 (A) and Rantes (B). Cell migration determined by fluorescence based assay, values expressed as units of fluorescence. Experiment included non-labeled and FePro labeled THP-1 cells incubated without the treatment (white bars), incubated in the presence of 10% FBS (grey bars) and 300 ng/ml of MCP-1 or 50 ng/ml of Rantes (black bars). Bars, means±SD. * p<0.05 compared to non-labeled control cells.

One of the important characteristics of the cells of macrophage/monocytic lineage is the ability to exhibit directional migration, i.e. chemotaxis, along the concentration gradients of biologically active, low molecular weight peptides called chemokines. Therefore we analyzed the effect of FePro labeling on THP-1 cells chemotaxis by determining their ability to migrate along the concentration gradients of MCP-1 and Rantes chemokines. When incubated for 4 h in the presence of MCP-1, FePro labeled THP-1 cells migrated in significantly smaller numbers compared to the non-labeled control cells (Figure 5A). Furthermore, this decrease in ability of FePro labeled cells to migrate was also observed when cells were incubated in the presence of 10% FBS. On the other hand, responsiveness of THP-1 cells to Rantes was not significantly affected by FePro labeling as no difference was observed between FePro labeled and non-labeled THP-1 cells (Figure 5B). These data indicate that FePro labeling may cause an overall decrease in THP-1 chemotaxis. However, it is possible that an unknown mechanism may be in place that enables the maintenance of chemotaxis towards Rantes after the FePro labeling.

### Effect of FePro labeling on THP-1 cells ability to modulate T cell function

**Figure 6:**
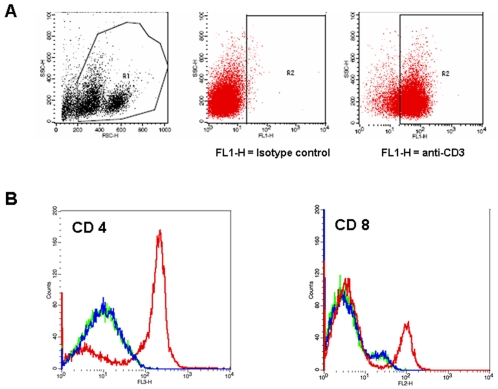
Effect of FePro labeling on THP-1 cell ability to modulate T cells. The data depicts the levels of CD markers expression after 24 h of THP-1 cell-T cell co-culture. T cells were stained with FITC conjugated anti-CD3, PE/Cy5 conjugated CD4 and PE conjugated anti-CD8. Cells were gated based on the forward and side scatter characteristics (R1) and on the expression of CD3 marker (R2) (A). Flow cytometric histograms of T cells cultured without the presence of THP-1 cells (red line), T cells co-cultured with non-labeled THP-1 cells (blue line) and T cells co-cultured with FePro labeled THP-1 cells (green line) from representative experiment are shown. THP-1 cells induced modulation of T cell expression levels of CD4 and CD8 (B). Note the complete overlap of blue and green lines. At least 10,000 live-gated cells were analyzed for CD4 and CD8 expression.

**Figure 7:**
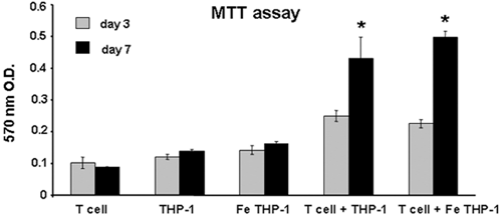
Effect of FePro labeling on THP-1 ability to induce T cell proliferation. Non-labeled and FePro labeled, gamma irradiated, THP-1 cells co-cultured with T cells for 7 days. MTT proliferation assay performed at day 3 (gray bars) and day 7 (black bars). Data are presented as a mean±SD of values of optical density measured at 570 nm wavelength. Statistical analysis was performed by comparing proliferation at day 7 with day 3 within the group (labeled or non-labeled THP-1 co-culture and labeled or non-labeled THP-1 cells only or T cells only) and by comparing proliferation at day 7 between the groups (labeled vs. non-labeled THP-1 co-culture). * = p<0.05 day 7 compared to day 3. No significant difference was observed between labeled and non-labeled conditions in co-culture and in THP-1 cells only samples.

As part of the “first line of defense” macrophages play a crucial role in innate immunity. However, as antigen presenting cells macrophages have an important function in adaptive immunity as well. One of the aspects of macrophage role in modulating adaptive immune response is the interaction with T cells. Therefore, we have analyzed the effect of FePro labeling on the THP-1 cells ability to modulate allogeneic CD3^+^ T cells by analyzing T cell surface expression of CD4 and CD8, and T cell proliferative activity. After 24 h hours of co-culturing T cells with THP-1 cells a significant decrease in T cell surface CD4 expression was observed, as compared to CD4 expression in control T cells (cells cultured without THP-1 cells). Detected CD4 expression in T cells co-cultured with THP-1 cells was lower then the basal expression observed in control T cells. More importantly, there was no difference in the levels of CD4 expression between T cells cultured with FePro labeled THP-1 cells and T cells cultured with non-labeled THP-1 cells (Figure 6B). THP-1 induced down-regulation of T cell surface CD8 expression was of less magnitude compared to the THP-1 induced down-regulation of CD4. However, the observed shift in expression was of the same pattern in T cells cultured with FePro labeled THP-1 cells and T cells cultured with non-labeled THP-1 cells (Figure 6B). MTT analysis revealed that THP-1 induced allogenic T lymphocyte proliferation was comparable in T cells co-cultured with non-labeled THP-1 cells and T cells co-cultured with FePro labeled THP-1 cells. Both groups exhibited significant increase in formazen production (MTT assay product) at day 7 of culture, with no significant difference between groups (Figure 7). These data indicate that FePro labeling did not affect the ability of THP-1 cells to stimulate proliferation in allogeneic T cell.

## Discussion

As immunologically active cells, through the activation of a multitude of signaling pathways and complex interaction with the other cell types, macrophages play a crucial role in both, the adaptive and acquired immune responses and the ability to respond to various stimuli is one of the major prerequisites for this physiological role of macrophage type cells. In the present study, we show that magnetic labeling with SPIO agent Ferumoxides-Protamine Sulfate (FePro) does not alter major immunological properties of macrophage-like THP-1 cells. As shown, THP-1 cells labeled with FePro method exhibited abundant uptake of iron and it has been postulated that this ferumoxides nanoparticles uptake in macrophages may be mediated *via* the scavenger receptor SR-A mediated endocytosis [25]. The present findings indicate that endocytosed FePro nanoparticles do not alter the ability of THP-1 cells to differentiate. Incubation of FePro labeled THP-1 cells with a differentiating agent TPA resulted in the expected changes in cell morphology as well as in increase in the cell surface expression of CD11b, CD117, CD86 and HLA-DR markers. Integrin α-M (CD11b), a protein subunit of the heterodimeric integrin αMβ2 complex expressed on the surface of cells involved in innate immunity [26], has been shown to play an important role in leukocyte activation, adhesion, migration and phagocytosis [27]. Stem cell factor receptor or c-KIT (CD117), a receptor tyrosine kinase type III, has been described to play a role in cell survival, proliferation and differentiation [28]. CD86, co-stimulatory protein necessary for activation and priming of T cells, has been reported to be expressed at low levels by monocytes and this expression is increased upon cell activation [29], [30]. Human Leukocyte Antigen DR (HLA-DR), a major histocompatibility complex class II receptor with the primary role of presenting (foreign) peptide antigens to the immune system, has also been described to be commonly expressed in macrophages [31]. Equal response to TPA in non labeled and FePro labeled THP-1 cells with regard to the expression of these cell surface molecules indicates that labeled cells preserved their capacity to differentiate into immunologically competent cells.

One of the pivotal roles of these immunologically active cells is to combat infection/injury with an immediate, non-specific reaction. Macrophages play a crucial role in this response by migrating towards the source of stimulation and by releasing cytokines that will further modulate the immunological response. As mentioned earlier, one of the stimuli that play a role as macrophage activator and that is released during Gram-negative bacterial infections is LPS. The pro-inflammatory response to LPS of macrophage like cells was characterized by an increase in TNF-α secretion and CD 54 and CD 83 cell surface expression, and is partially mediated by NFκB cell signaling pathway [17]. Intracellular Adhesion Molecule One (ICAM-1 or CD54) was previously shown to be up-regulated in response to a variety of inflammatory factors and to play a role in leukocyte migration and T cell activation [24]. CD83 is expressed by mature dendritic cells (DCs) as well as in DCs that were *in vitro* derived from monocytes [32], and it is also postulated to play a role in activation of T-cells [33]. Here, we show that FePro labeling did not affect THP-1 cells ability to upregulate CD54 and CD83 in response to LPS. In addition, increase in THP-1 cell's TNF-α expression in response to LPS stimulation was also not affected by FePro uptake. The observed preserved ability of iron labeled THP-1 cells to secrete TNF-α in response to LPS contrasted the results reported by Siglienti et al., that indicated the inhibitory effect of iron uptake on TNF-α production in LPS stimulated, iron labeled, rodent resident peritoneal macrophages [34]. Since previous work by different groups showed that resident macrophages isolated from rodent peritoneal cavity release minimal amounts of TNF-α in response to LPS-stimulation [35], [36], it is possible that this discrepancy is due to differences in the species and the type of cells used in the studies. Interestingly, western blot analysis of nuclear p65 NFκB and cytoplasmic IκB levels showed that THP-1 intracellular FePro incorporation may cause this pathway to be up-regulated. Previous studies have shown that cells labeled with iron oxide nanoparticles complexed to cationic transfection agent exhibited transient increase in reactive oxygen species (ROS) [10]. In addition, increase in the intracellular ROS production has been shown to activate NFkB pathway as well as increase the expression of NFkB p65 subunit [37], [38]. However, no difference in the levels of TNF-α secretion and CD54 and CD83 cell surface expression between FePro labeled and non labeled THP-1 cells indicated that the observed slightly higher nuclear p65 NFκB protein levels (accompanied with expected changes in the cytoplasmic IkB levels) in FePro labeled THP-1 cells did not have significant effect at the level of protein expression.

Another important property of macrophage cell type is the ability to migrate along the concentration gradient of various biomolecules. This chemotactic ability is necessary for the *in vivo* regulation and control of inflammation. Rantes and MCP-1 are molecules that belong to the C-C class of the beta chemokine supergene family and are implicated to play a central role in regulating acute as well as chronic inflammatory processes by mediating the migration of mononuclear and polymorphonuclear leukocytes [39], [40]. Experiments presented here showed differential response of FePro labeled THP-1 cells towards MCP-1, Rantes and serum proteins. Overall decrease in chemotaxis towards MCP-1 and serum proteins indicate possible nonspecific effect of the iron load on the ability of THP-1 cells to migrate. However, the same effect was not observed when THP-1 cells were incubated in the presence of Rantes, i.e no difference was observed between the migration of FePro labeled and non-labeled THP-1 cells. Previous studies done on Rantes and other chemokines indicated that mononuclear and other cells express promiscuous receptors that recognize one or more chemokine within the beta family and this phenomenon maybe influenced by differences in the binding affinities between different receptors and chemokines [41], [42]. It is possible that mechanisms of the observed FePro effect on THP-1 chemotaxis involve changes in the expression and/or function of chemokine receptors. However, to further evaluate the implication of the observed altered THP-1 chemotaxis on the clinical application of FePro labeling, more complex experimental design that would include the sequential blockage of the receptors under the investigation is warranted. Nevertheless, the *in vitro* set up presented here provided the general insight on the existence of chemokine dependent, differential effect of FePro nanoparticles on THP-1 cell chemotaxis.

In addition to their important role in innate immunity, macrophages play a role in adaptive immunity [43] and one of the aspects of this function is modulation and interaction with T cells. Data presented here show that THP-1 cells co-cultured with T cells induced a decrease in T cell expression of CD4 or CD8 cell surface markers that define helper and cytotoxic T cell population, respectively. The ability of THP-1 cells to down-regulate CD4 and CD8 on T cells was not affected by FePro labeling. CD4 and CD8 play a role as T cell receptor co-receptors (TCR) that ensure the binding specificity of TCR for a specific antigen and enhance the signal initiated through TCR complex. The concrete mechanisms of CD4 and CD8 downregulation have not been described before. It has been previously shown that macrophages may be involved in CD4+ T helper cell depletion [44]. However, the observed downregulation may also involve IL-1 and/or INF-γ mediated effects [45], [46]. Another aspect of macrophage interaction with T cells is antigenic presentation and T cell activation and one of the features of this interaction is induction of T cell proliferation. Here we show that T cells proliferative activity in response to THP-1 cells was not affected by FePro labeling of THP-1 cells. This data indicate that iron labeling may not have negative effect on macrophage role as antigen presenting cells.

With further development of cell transplantation based imaging techniques and correlated iron oxide cell labeling techniques it is of utmost importance to ensure for minimal side effects that may be caused by macrophage mediated *in vivo* clearance of administered iron. The data presented here, generated from an *in vitro* experimental set up, give the general insight into the possible effects of intracellular FePro accumulation. However, it is important to emphasize that under the *in vivo* conditions immunological processes are the result of complex interplay among tissue resident immune cells, cells recruited from circulation, cytokines and other molecules generated in the course of a variety of pathological processes. Therefore, the effects of FePro on macrophage modulatory role may necessitate an *in vivo* set up that would enable the analysis of the ability of immune system to respond to different pathological challenges. In summary, by utilizing THP-1 cell line as a model for macrophage cell type and by measuring a variety of endpoints (cell differentiation, cell surface marker expression, cytokine production, chemotaxis and immunological modulation) we demonstrated that endocytosed FePro nanoparticles did not significantly affect immunological properties of THP-1 cells.
